# Root Migration After Coronectomy of a Mandibular Third Molar With the Inferior Alveolar Nerve Passing Between the Roots: A Case Report

**DOI:** 10.1002/ccr3.73673

**Published:** 2026-10-12

**Authors:** Vito Marquez Rocco, Andreas Thor, Abdullah A. Hashim, Dominique Bellerud

**Affiliations:** ^1^ Department of Oral and Maxillofacial Surgery Aalborg University Hospital Aalborg Denmark; ^2^ Karolinska Institutet Stockholm Sweden; ^3^ Department of Surgical Sciences Uppsala University Uppsala Sweden; ^4^ Department of Plastic and Oral and Maxillofacial Surgery Uppsala University Hospital Uppsala Sweden; ^5^ Department of Oral and Maxillofacial Surgery Västerås Hospital Västerås Sweden

**Keywords:** coronectomy, inferior alveolar nerve injury, mandibular third molar surgery, oral surgery, root migration

## Abstract

Extraction of mandibular third molars (MTMs) is one of the most common procedures in oral and maxillofacial surgery. It carries a risk of inferior alveolar nerve (IAN) injury, which may result in altered or lost sensation of the lower lip, chin, teeth, and gingiva. Coronectomy, a surgical technique in which the crown is removed while the vital roots are retained, has been proposed to reduce this risk in cases with close root–nerve proximity. We report the case of a 41‐year‐old female patient in whom cone beam computed tomography (CBCT) revealed the IAN coursing between the roots of an MTM. Coronectomy was performed to preserve nerve integrity. Follow‐up CBCT demonstrated significant migration of the retained root complex within 6–12 months, increasing the distance from the IAN. Five years later, infection associated with the retained roots required surgical root removal. Owing to the favorable migration of the root complex, extraction was completed without IAN injury. This case illustrates that, despite the possibility of late complications requiring reintervention, coronectomy may facilitate root migration away from the nerve and allow safer delayed root removal in anatomically high‐risk situations. Careful patient selection, meticulous surgical technique, and long‐term radiographic follow‐up are essential. Further high‐quality long‐term studies are needed to standardize coronectomy protocols, evaluate complications, and assess cost‐effectiveness compared with conventional extraction.

## Key Clinical Message

Coronectomy may reduce the risk of inferior alveolar nerve injury in high‐risk mandibular third molars. Although complications and secondary surgery may occur, postoperative root migration can increase root–nerve separation, facilitating safer delayed root removal. Careful case selection, surgical technique, and long‐term follow‐up are essential.

## 1 Introduction

The most frequent surgical procedure in oral surgical practice is the extraction of the mandibular third molar (MTM) [1, 2, 3, 4]. This treatment is associated with well‐known intraoperative and postoperative risks, such as pain, edema, trismus, hemorrhage, displacement of the extracted tooth into adjacent spaces, mandibular angle fracture, infection and injury to the inferior alveolar nerve (IAN) [1, 5]. Regarding the IAN, postoperative affections can include altered or loss of sensation of the lower lip, chin, teeth and gingiva on the operated side. This can be perceived by the patient as tingling, numbness, or a burning or painful sensation, potentially resulting in a reduced quality of life. The prevalence of IAN injury with temporary effect goes from 0.35% to 8.4% [1, 2, 3, 4, 5, 6, 7, 8, 9, 10, 11, 12]. The incidence may reach 19%–50% in high‐risk situations [1, 2, 5, 9, 12]. Permanent nerve injury occurs between 0.014% and 3.6% of these cases [1, 2, 4, 5, 8, 9, 12].

Coronectomy of MTMs, also known as partial odontectomy, was first reported by Ecuyer and Debien in 1984. It is described as a procedure in which the vital roots are intentionally and preoperatively planned to be left in the mandibular bone after removal of the crown (see Table 1 for an overview of the surgical steps). This procedure primarily aims to mitigate the risk of IAN injury when the risk is identified radiologically [1, 2, 6, 10, 15, 16].

**TABLE 1:** Surgical technique of coronectomy.

Step	Description
1. Surgical access	Raise a full‐thickness mucoperiosteal flap and perform osteotomy down to the cementoenamel junction (CEJ)
2. Crown sectioning	Section the crown 1–2 mm below the CEJ to two‐thirds or three‐quarters of its depth
3. Crown removal	Carefully remove the crown using a luxator to avoid disturbing the root complex
4. Enamel removal	Use fissure or round burs to eliminate any remaining enamel
5. Root position verification	Ensure roots are located at least 3–4 mm below the crest of the buccal and lingual cortical plates
6. Pulp management	Leave the pulp untreated
7. Wound debridement	Thoroughly irrigate and clean the surgical site with saline
8. Wound closure	Achieve primary closure of the flap using sutures

*Note:* References: [2, 5, 7, 8, 10, 12, 13, 14].

### 1.1 Migration of the Left Root After Coronectomy

The retained root's migration after coronectomy of MTM is a frequently observed postoperative phenomenon, and is generally considered a positive consequence that occurs in 84%–97% of cases [4], with a mean migration distance of 2.33–5.27 mm within the first 6–12 months [3, 4, 5, 7, 8, 9, 11, 16, 17, 18]. The frequency and extent of root migration reportedly reach the highest level 6 months after coronectomy and gradually decrease as bone grows over the retained root [15]. Most roots show a tendency to stop migrating toward the end in follow‐up studies of 2–3 years following the surgical intervention. Some studies suggest that migration distance is negatively correlated with age, meaning that it is more pronounced in younger patients. It is also described that it has a greater extent in females [4, 7].

Given its effectiveness in preserving nerve function and reducing postoperative complications, coronectomy can be considered a surgical approach, when properly indicated, for managing retained MTMs with high risk of IAN injury. However, the method should be avoided in certain conditions such as infection, mobility of the root remnants, or incomplete removal of enamel (see Table 2 for a summary of contraindications). Moreover, root migration is a common postoperative event that can be considered a positive event, and it rarely leads to adverse outcomes, making the procedure a safe and viable alternative to total tooth extraction [2, 18].

**TABLE 2:** Contraindications of coronectomy.

Contraindication	Explanation
Deep caries, endodontic infection, or periodontal infection	Infected teeth may impair healing or cause postoperative complications
Inability to remove all enamel	Remaining enamel may lead to eruption or cyst formation, resulting in procedure failure
Excessive mobility of the retained root	Mobile roots may migrate or become symptomatic, requiring reoperation
Horizontally impacted tooth along the IAN	Sectioning in such cases carries a high risk of damaging the inferior alveolar nerve (IAN)
Teeth associated with cystic lesions or tumors	Such pathology often requires complete extraction rather than root retention, as spontaneous resolution is unlikely
Medically compromised patients	Retained roots may serve as an infection source, especially risky in these patients prior to chemotherapy or radiation

*Note:* References: [2, 5, 7, 8, 10, 12, 13, 14, 19, 20, 21].

Here, we present a case where root migration of the left MTM occurred in a significant way regarding diminishing the IAN risk after coronectomy despite the nerve being positioned in between the root complex and markedly compressed.

## 2 Case History/Examination

A 41‐year‐old woman was referred to the Department of Oral and Maxillofacial Surgery at Uppsala University Hospital for evaluation of the left mandibular third molar. Her medical history included mild asthma; she reported no known allergies and was a non‐smoker. She was referred because of recurrent pericoronitis associated with the left mandibular third molar.

Clinical examination revealed a partially erupted left mandibular third molar with tenderness on buccal palpation. A 6‐mm distobuccal periodontal pocket with purulent discharge was present. No trismus was observed, and examination of the neck and cervical lymph nodes was unremarkable.

Preoperative panoramic radiography (Figure 1) demonstrated a partially erupted and distally inclined tooth 38 with an enlarged pericoronal space. The roots appeared compact and overlapped the mandibular canal. CBCT (Figure 2, series 1) showed an enlarged pericoronal space and complex root morphology consisting of four anatomical roots: three lingual roots (two mesiolingual and one distolingual), fused apically into a single lingual root complex, and one buccal root. The mandibular canal coursed between the roots and appeared compressed by them (Figure 1, series 1 and Figure 3).

**FIGURE 1:**
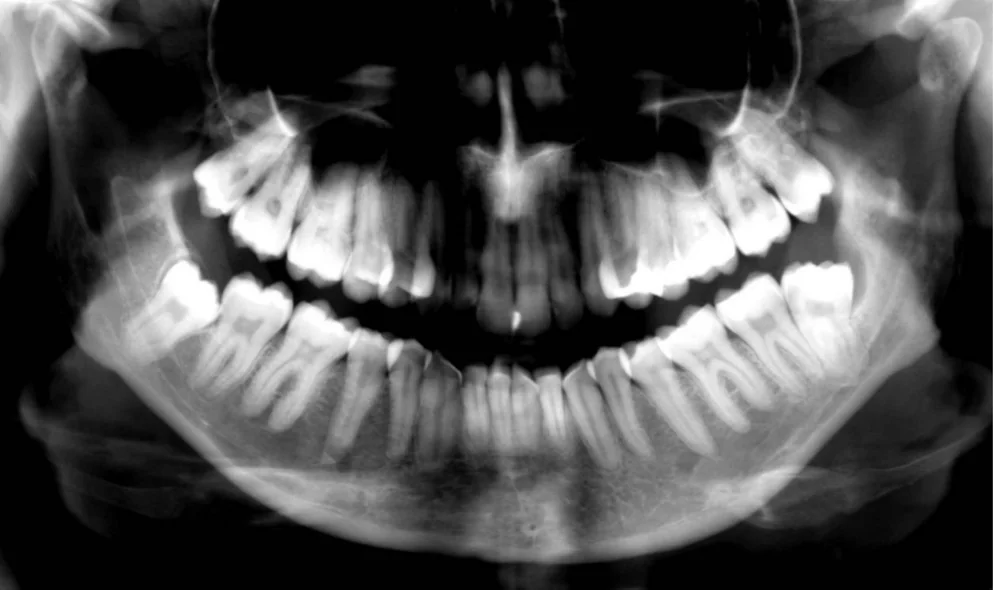
Panoramic x‐ray pre‐coronectomy.

**FIGURE 2:**
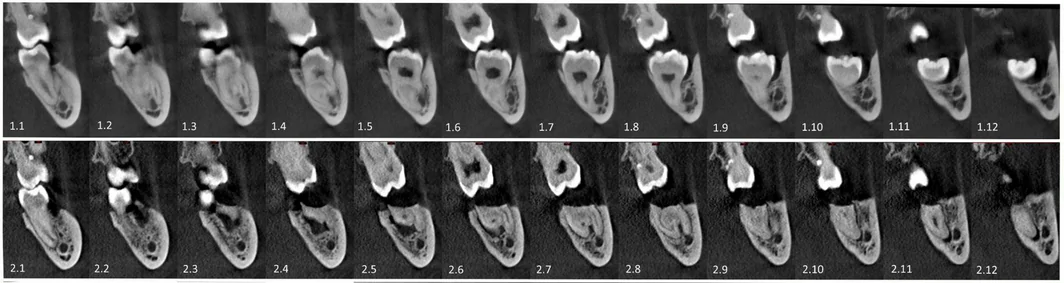
Comparison of sagittal cuts pre‐coronectomy and post‐coronectomy, demonstrating migration of the root complex after the procedure. Top series: Pre‐coronectomy. Bottom series: Post‐coronectomy.

**FIGURE 3:**
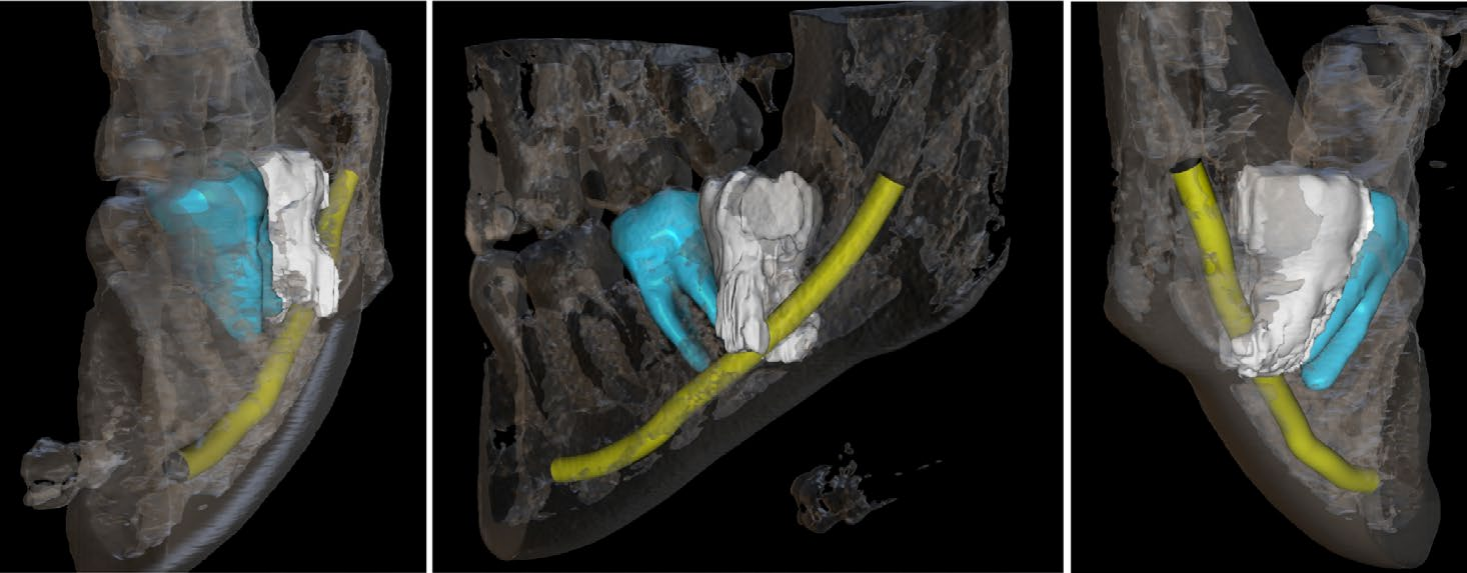
Representative segmentation from CBCT‐scan prior to coronectomy. Anterior, lateral, and posterior view from left to right.

## 3 Diagnosis, Investigations, and Treatment

The diagnosis was chronic pericoronitis associated with semi‐retained tooth 38. Given the close relationship between the tooth roots and the IAN, complete extraction was considered to carry a high risk of nerve damage.

Based on the clinical and radiological findings, coronectomy was selected to minimize the risk of IAN injury. The patient was informed about the procedure and possible complications, and informed consent was obtained.

The coronectomy was performed in May 2019, under local anesthesia and light conscious sedation with oral midazolam (Midazolam APL, Apotek Produktion & Laboratorier AB, Stockholm, Sweden; 1 mg/mL oral solution), administered approximately 1 h before surgery at a dose of 10 mg (10 mL). A buccal flap was raised, and the crown was sectioned and removed in three fragments. The roots were reduced to a level below the alveolar bone crest. The surgical site was cleaned, irrigated, and closed using Vicryl Rapide 4‐0 sutures (Ethicon Inc., Somerville, NJ, USA).

At the 2‐week postoperative follow‐up, no signs of infection were observed. A gingival dehiscence with food impaction was noted, and the patient was instructed to perform local irrigation with saline solution. A postoperative panoramic radiograph demonstrated the retained roots of tooth 38 following coronectomy. A postoperative panoramic radiograph (Figure 4, left side) demonstrated the retained roots of tooth 38 following coronectomy with a horn of enamel tissue medially.

**FIGURE 4:**
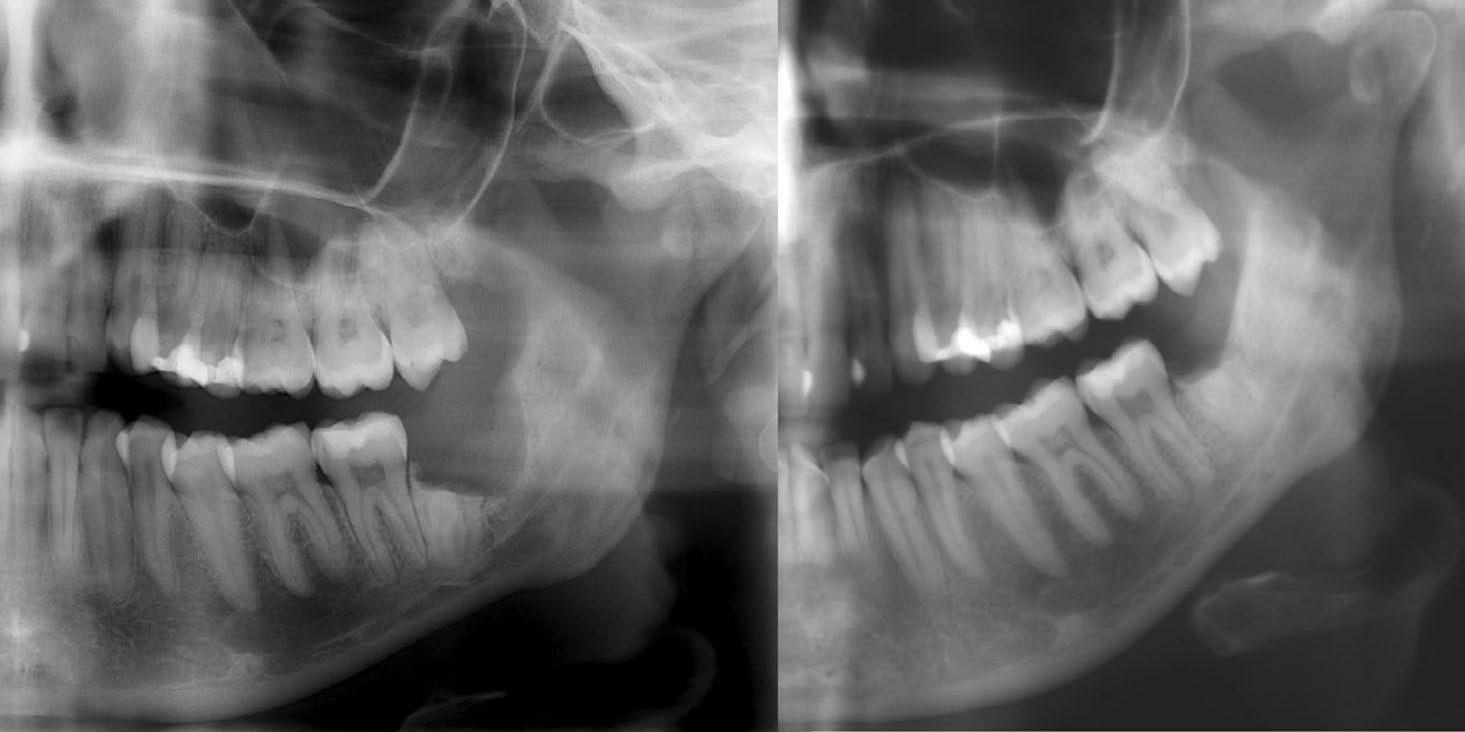
On the left side: Immediate post‐coronectomy panoramic x‐ray. On the right side: 4,5 years post‐coronectomy. It can be clearly noted how the root complex has migrated upwards, backwards and rotated slightly anteriorly.

At the 14‐month follow‐up, the patient was asymptomatic. A minor mucosal opening of approximately 1 mm persisted, and radiographic examination (Figure 4, right side) showed approximately 2 mm of root migration without associated pathology. Further follow‐ups in August 2020 and January 2024 revealed that the patient remained largely symptom‐free, reporting only persistent food impaction. No purulence, deep periodontal pockets, or radiographic abnormalities were identified.

Five years after the coronectomy, the patient developed pain and symptoms resembling recurrent pericoronitis, including local swelling, purulent discharge, and fistula formation (noticed in Figures 5 and 6). Initial management consisted of local irrigation and the patient was prescribed phenoxymethylpenicillin (Kåvepenin, Karo Healthcare AB, Stockholm, Sweden) 1.6 g three times daily for 7 days. A CBCT (Figure 2, series 2) revealed signs of infection associated with the left root complex of the tooth 38 in the form of periodontal bone loss on the mesial aspect and apical periodontitis. The CBCT also demonstrated superior migration of the root complex, now positioned above the mandibular canal. The IAN was located inferior to the root apices (Figure 2, series 2 and Figure 7).

**FIGURE 5:**
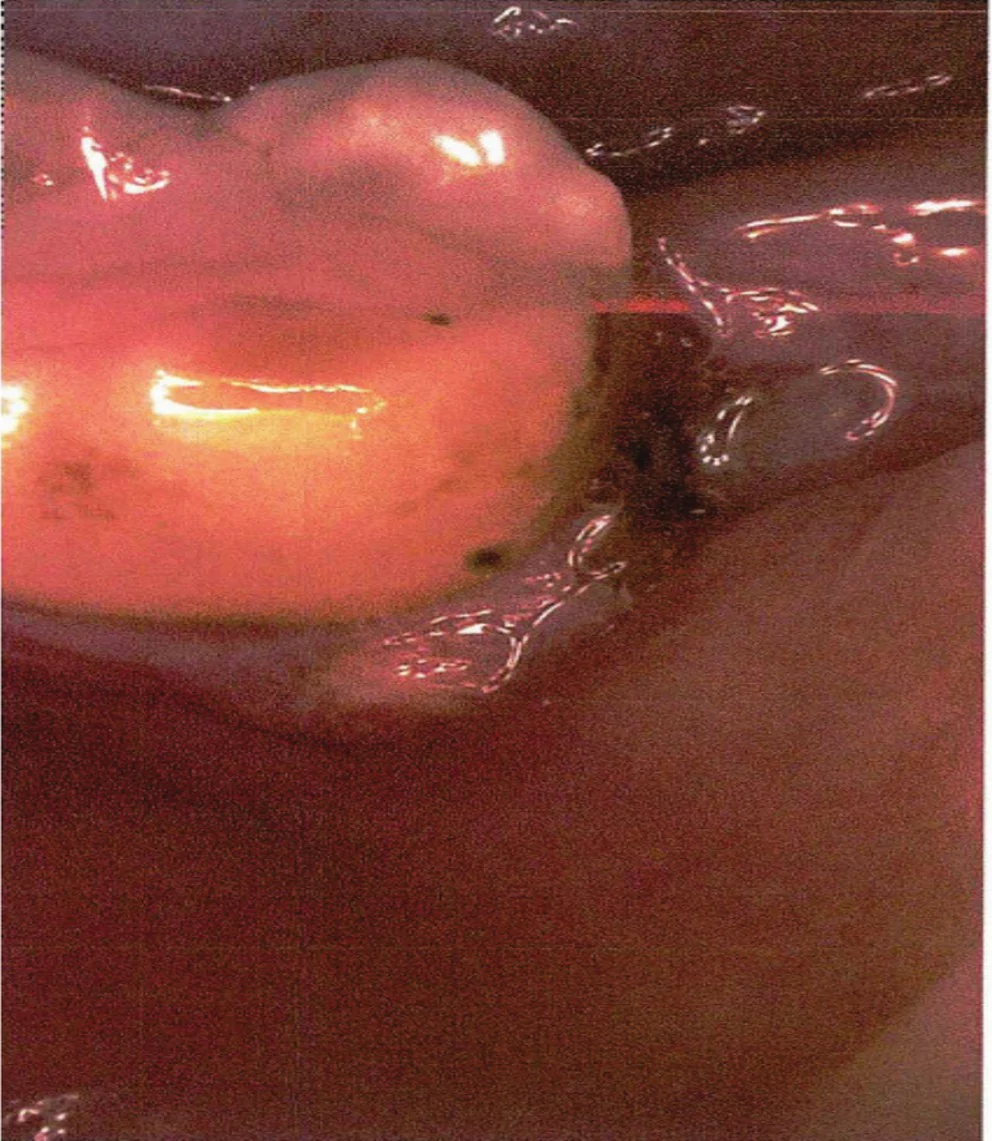
Fistula with pus distobuccal of tooth 37 at the second referral.

**FIGURE 6:**
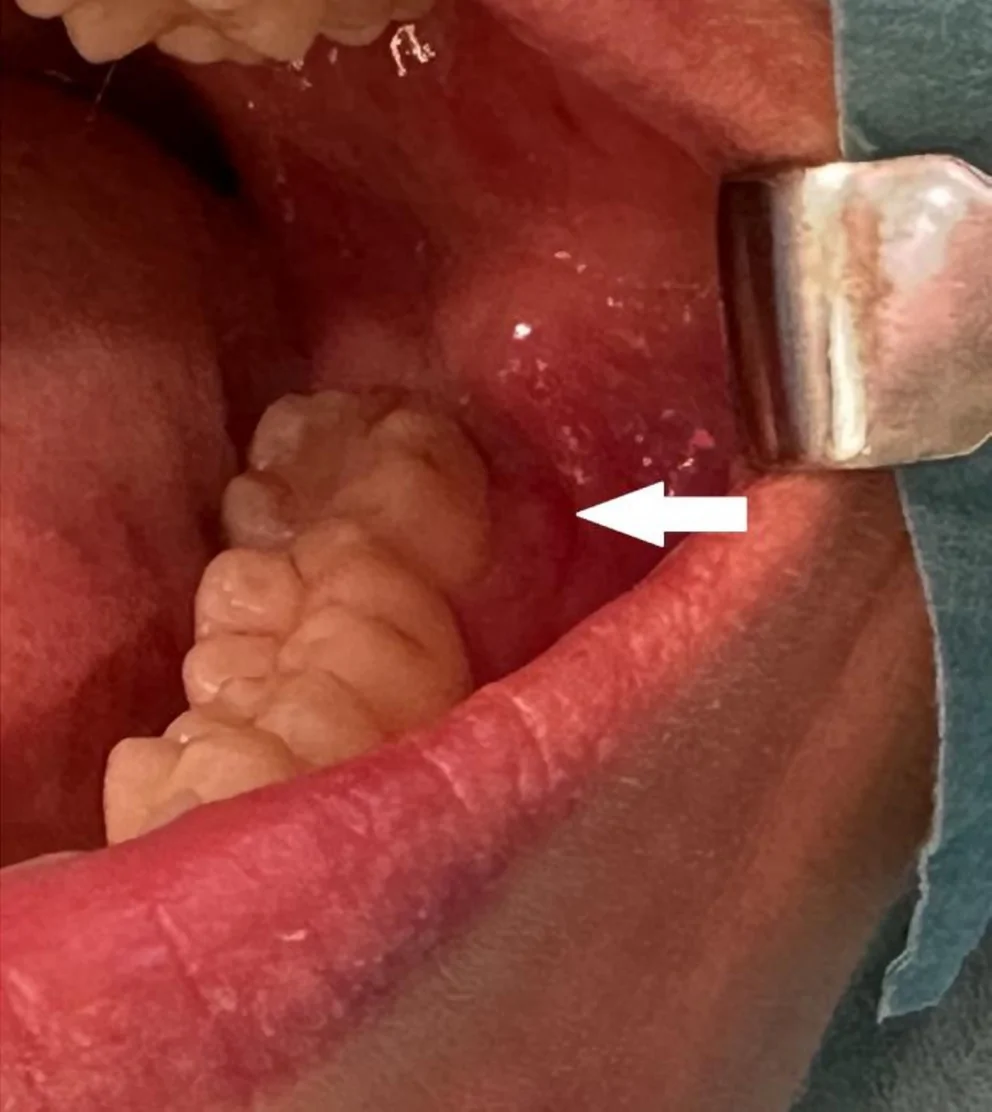
Fistula distobuccal of tooth 37 just prior to surgical removal of left root complex of tooth 38.

**FIGURE 7:**
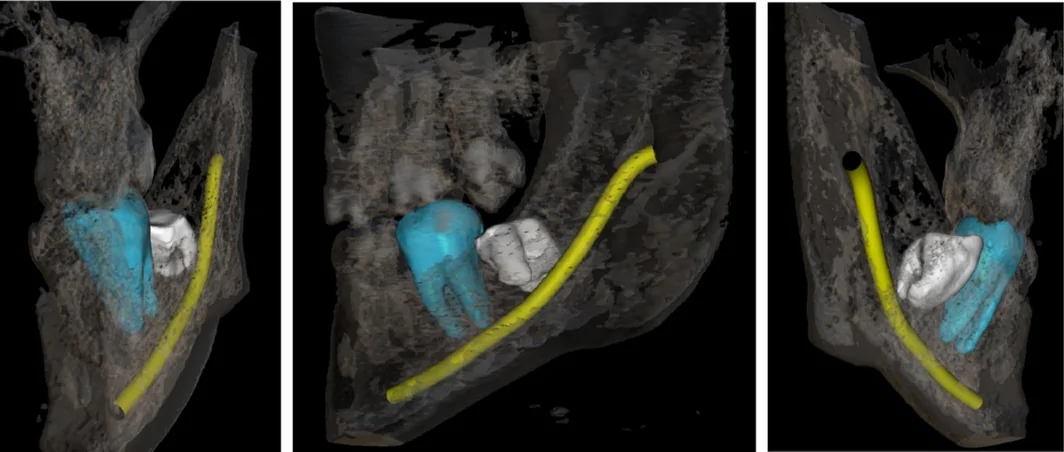
Representative segmentation from CBCT‐scan post decoronation. Anterior, lateral, and posterior view from left to right.

Given these findings, surgical removal of the retained roots was indicated. The procedure was performed in April 2025, under the same protocol of light sedation and local anesthesia. A buccal flap was raised extending from the region of teeth 37 to 38 (Figure 8). After buccodistal osteotomy, the root complex was removed. The fused lingual root complex was retrieved as one specimen, whereas the buccal root fractured during extraction and was removed in two separate fragments, representing the original four anatomical roots (Figures 9 and 10). Alveolar curettage, bone smoothing, and copious irrigation were performed, followed by primary closure with Vicryl Rapide 4‐0 sutures (Ethicon Inc., Somerville, NJ, USA). Hemostasis was achieved.

**FIGURE 8:**
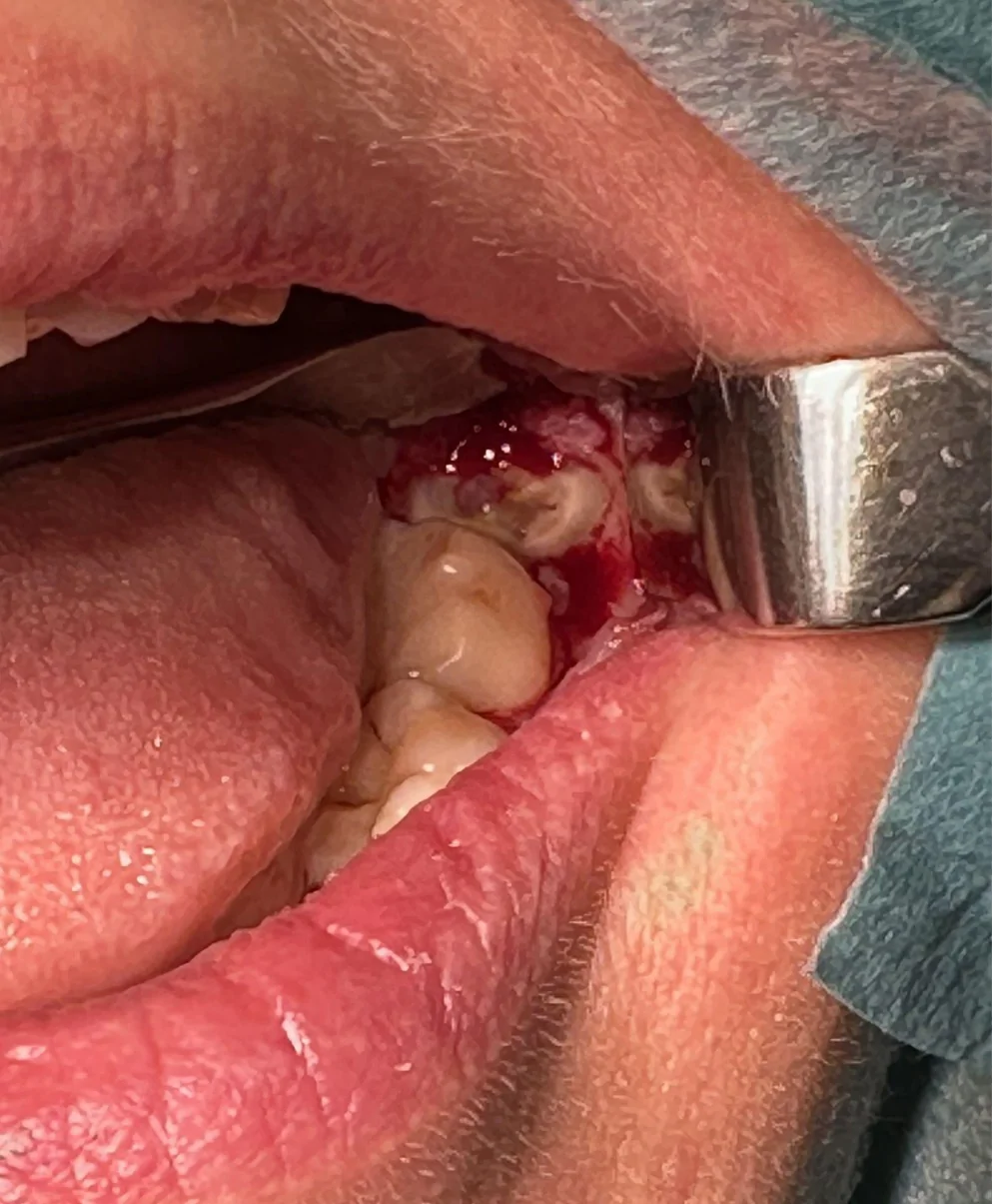
Intraoperative image of elevated buccal flap and exposed left root complex of MTM 38.

**FIGURE 9:**
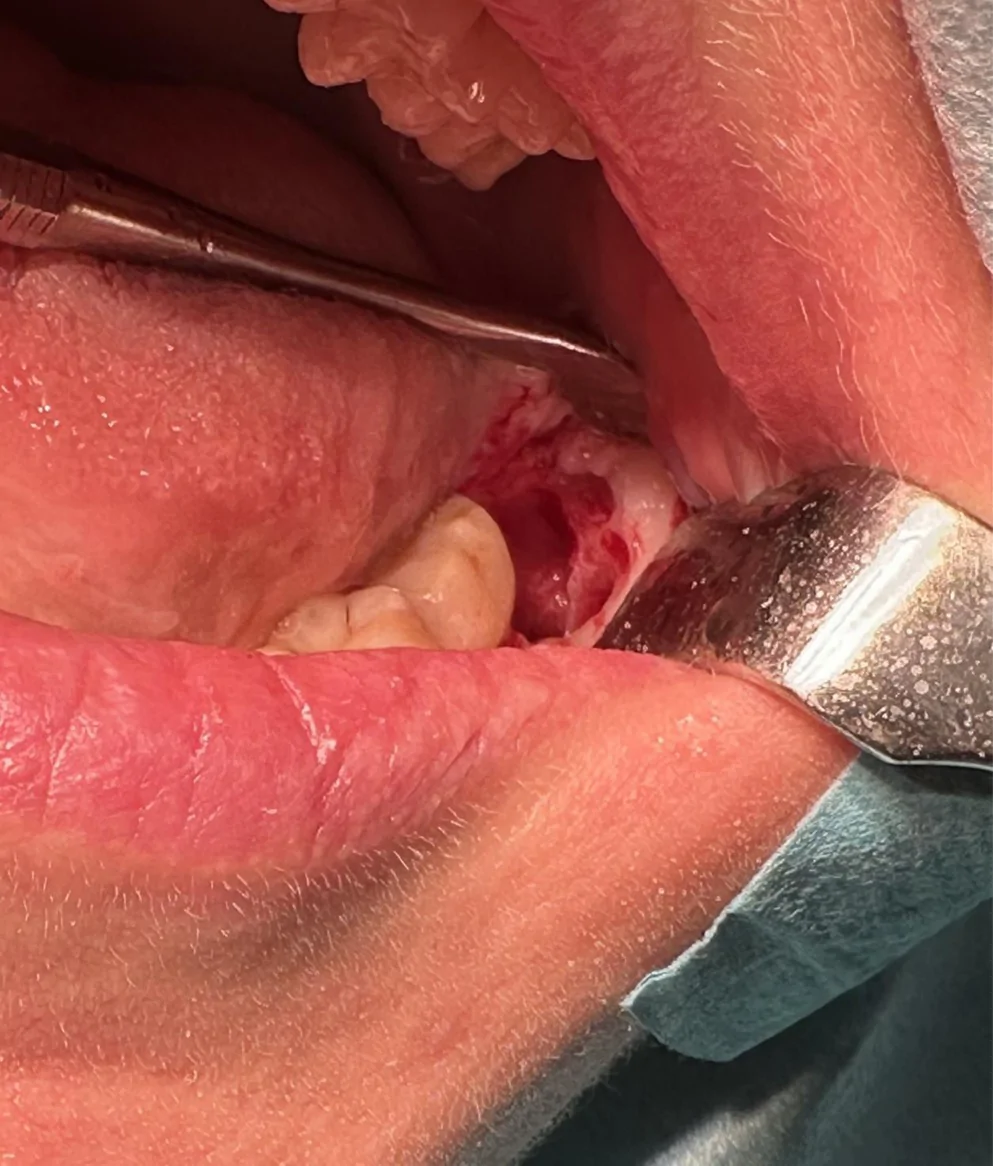
Extraction alveolus of 38. It can be seen that as the root complex migrated, there was bone formation in the interradicular area despite infection.

**FIGURE 10:**
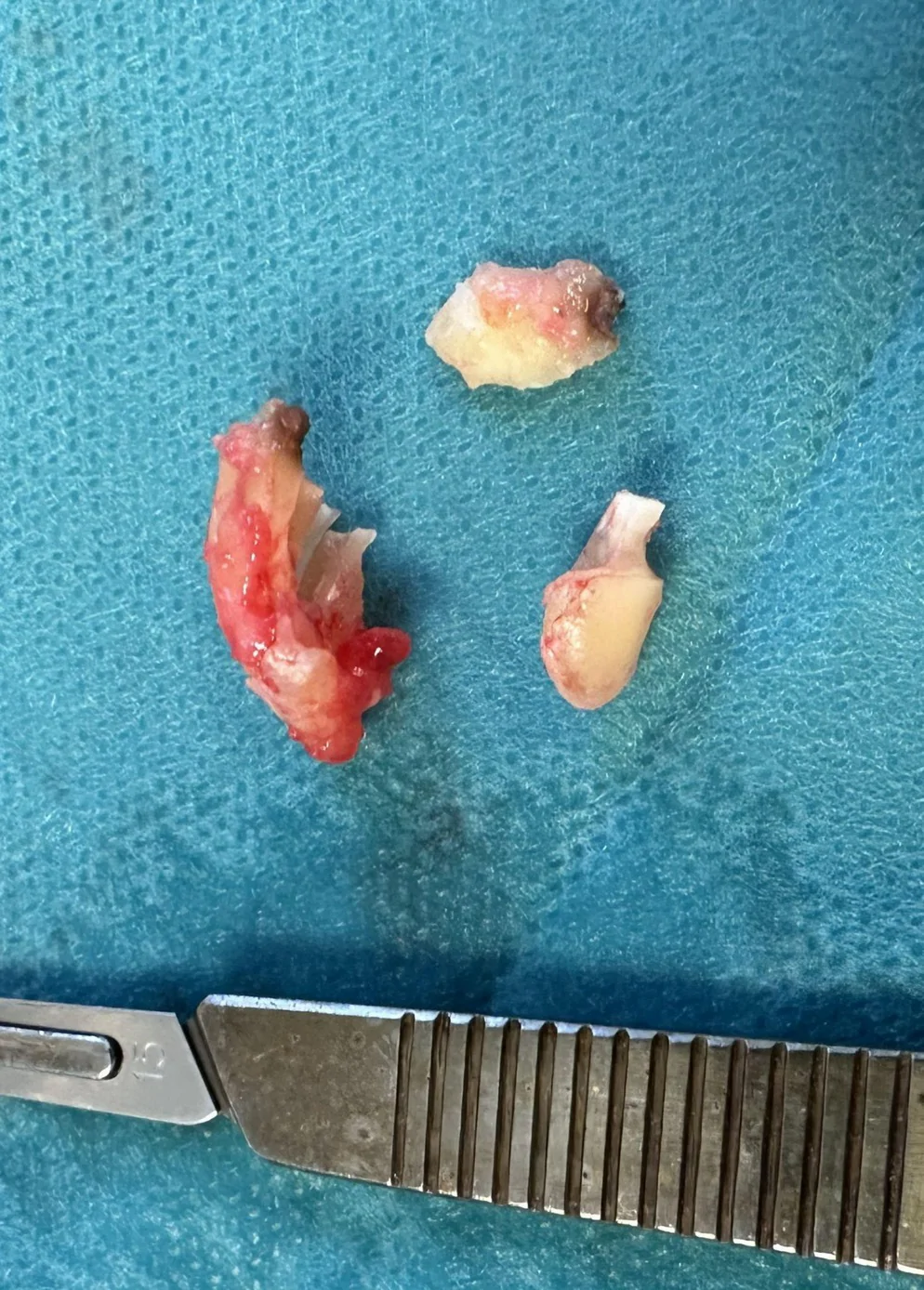
Extracted specimens after delayed root removal. The mandibular third molar originally consisted of four anatomical roots: three lingual roots fused into a single lingual root complex (left) and one buccal root (right). During extraction, the buccal root fractured and was retrieved as separate cervical and apical fragments.

## 4 Results and Conclusion (Outcome and Follow‐Up)

Postoperative follow‐up at 2 and 4 weeks after root removal demonstrated uneventful healing. Good primary closure of the surgical site in the region of tooth 38 was confirmed after 2 weeks, and no inferior alveolar nerve‐related complications were observed. A panoramic radiograph obtained at 4 weeks showed no evidence of residual pathology (Figure 11).

**FIGURE 11:**
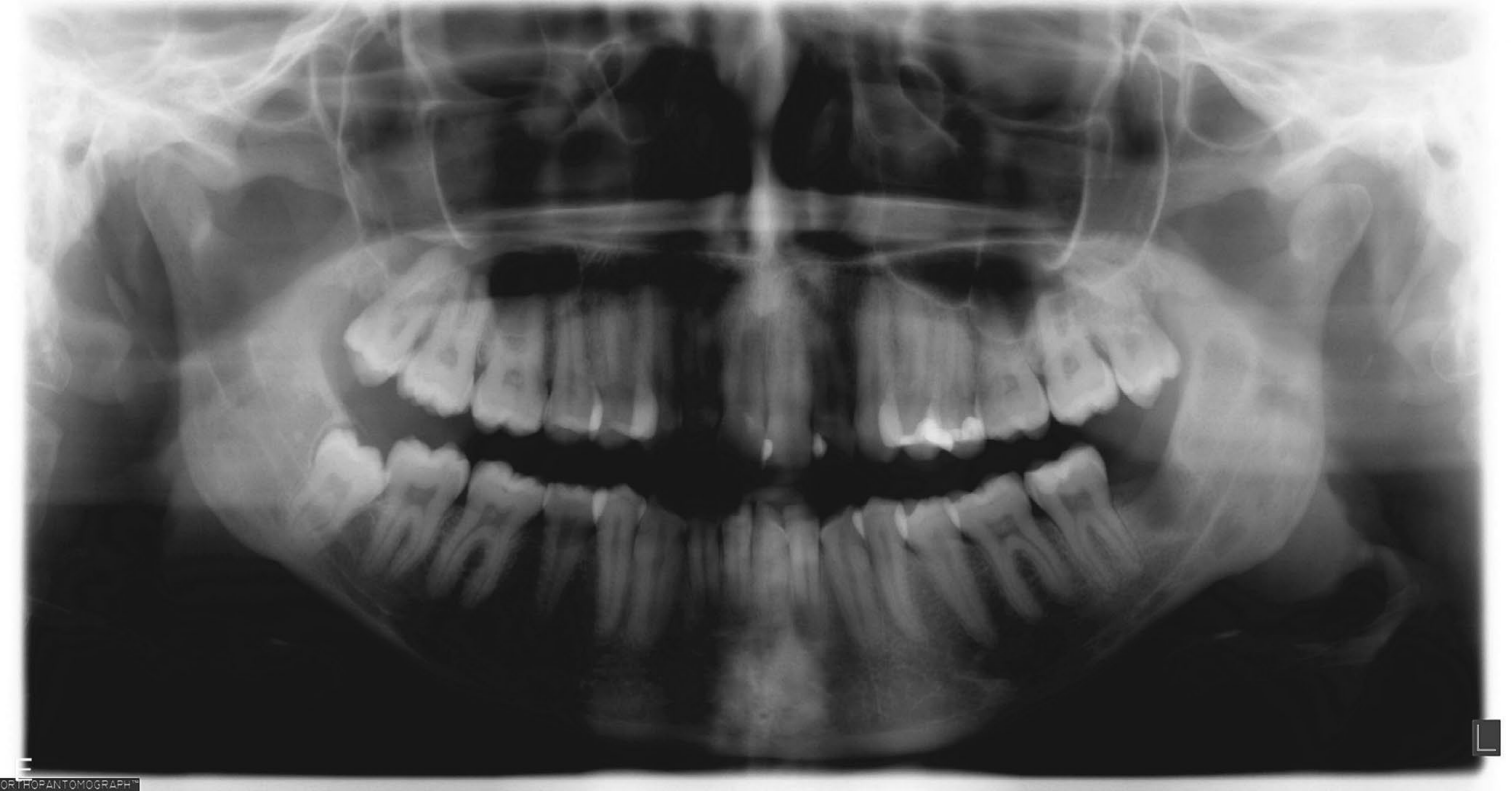
Post‐operative panorama x‐ray (4 weeks after) following removal of the root complex of the tooth 38.

This case illustrates that coronectomy of mandibular third molars may be an effective nerve‐preserving strategy in anatomically high‐risk situations. However, late complications requiring reintervention may occur, emphasizing the importance of meticulous surgical technique, individual risk–benefit assessment, and long‐term follow‐up.

Although root migration is commonly observed within the first 6–12 months after coronectomy and is generally considered a favorable biological response, secondary infection may develop several years after the initial procedure. Long‐term follow‐up is therefore essential, and delayed surgical removal of the retained root complex may be performed more safely once it has migrated away from the inferior alveolar nerve, when clinically indicated.

Successful outcomes depend on careful patient selection, precise surgical technique, and well‐planned clinical and radiological follow‐up. Further high‐quality long‐term studies are needed to better understand the outcomes of coronectomy, establish evidence‐based follow‐up protocols, and identify factors influencing root migration and long‐term complications.

## 5 Discussion

Coronectomy has emerged as a valuable alternative to conventional extraction in the management of retained MTMs closely associated with the IAN [1, 2, 3, 6, 7, 9, 12]. The primary advantage of this procedure is the possibility to significantly reduce the risk of an IAN injury in cases of close root‐nerve proximity as visualized radiologically [1, 2, 3, 6, 7, 9, 12]. In the present case, the IAN passed between the roots of the MTM. According to Kim et al. [22], the prevalence of the anatomical variant where the nerve passes between open roots is 1% and 0.4% between closed roots of the mandibular third molars.

A consistent finding across studies is that root migration is a common and expected postoperative consequence of coronectomy. The average migration distance ranges between 2 and 4 mm, with the majority of movement occurring within the first 6–12 months postoperatively [2, 3, 4, 17, 18, 23]. This was observed in the present case. By comparing the preoperative and postoperative CBCT scans, root migration of 4.44 mm superiorly, 2.27 mm distally, and 0.20 mm lingually was observed. As a result, the shortest distance between the retained root complex post‐coronectomy and the IAN was 0.71 mm, measured from the anatomical apex of the lingual root complex to the nerve (Figures 2, 3 and 7). In this way, the remaining root became significantly separated from the IAN, diminishing the risk of IAN damage during reoperation. To our knowledge, no previous report has described root migration in such a close anatomical relationship between the tooth and the IAN. The IAN can be found between the roots of the MTM in 0.9%–1.39% of cases [23, 24]. It would be clinically relevant to determine if there is significant predictability in the distancing between the root complex and the IAN after coronectomy in cases such as ours. Further studies are needed to determine whether similar root migration patterns could support coronectomy in comparable high‐risk cases.

In the present case, coronectomy was performed despite the tooth presenting recurrent pericoronitis, a condition that has been described as a relative contraindication to coronectomy in several publications [5, 13, 14, 19, 20, 21, 25, 26, 27]. However, the exceptionally intimate relationship between the root complex and the IAN, which was shown to pass between the roots, was considered to represent a substantial risk of nerve injury if complete extraction had been attempted. Nevertheless, the pre‐existing inflammatory condition may have contributed to the late infection.

Regarding infection related to the retained roots after coronectomy, Goto et al. [15], Martin et al. [8], and Pitros et al. [12] reported that root exposure and infection occur at relatively low rates (0.6%–6.8%), with retained enamel and inadequate crown removal identified as important risk factors for postoperative complications [2, 3, 8, 11]. In fact, the main indications for reintervention following coronectomy are root exposure, infection, and postoperative pain [2, 7, 8].

Another limitation of this report is that the soft tissue removed during the procedure was not submitted for histopathological examination, but it was clinically interpreted as inflammatory granulation tissue. Consequently, the possibility of an undiagnosed cystic lesion cannot be completely excluded. Theoretically, it could be considered as another contraindication for coronectomy, according to the literature. However, in our assessment, the risk of permanent neurological injury associated with complete extraction may outweigh the potential risk of postoperative complications related to left roots, supporting the importance of an individualized risk–benefit assessment.

In the present case, postoperative CBCT imaging (Figure 2) and clinical examination (Figure 12) demonstrated an important technical mistake, the presence of a mesial enamel horn adjacent to the second molar. This finding suggests that complete enamel removal may not have been achieved during the coronectomy procedure. Previous studies have highlighted retained enamel and incomplete crown reduction as factors associated with delayed soft tissue healing, root exposure, and secondary infection [2, 3, 7, 8, 11]. The persistence of this enamel remnant may therefore represent a contributing factor of the development of infection through impaired soft tissue coverage and subsequent bacterial contamination of the retained root complex. Furthermore, the soft tissue defect and fistula shown in Figure 6 were located precisely in the area where the enamel remnant was identified. Consequently, this case underlines the importance of strict adherence to established coronectomy protocols, particularly complete enamel removal and adequate reduction of the retained root complex below the alveolar crest.

**FIGURE 12:**
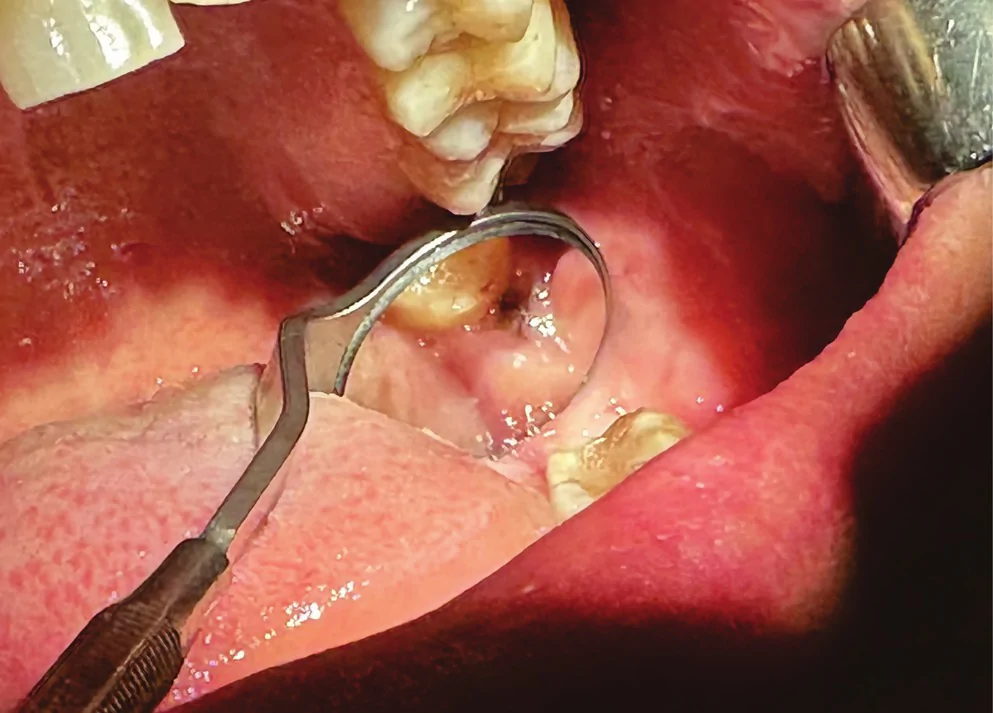
Residual mesial enamel horn of tooth 38, visible through a gingival dehiscence.

Importantly, the root migration observed in this patient must be carefully interpreted, since there were technical shortcomings in the coronectomy procedure. However, as mentioned in the introduction, root migration is a well‐documented and expected biological response after coronectomy, occurring in 84%–97% of cases and generally considered a favorable outcome because it increases the distance between the retained roots and the IAN [3, 4, 5, 7, 8, 9, 11, 16, 17, 18]. In the present case, migration of the root complex ultimately allowed surgical root removal to be performed in a safer anatomical position, reducing the risk of nerve injury during the second intervention.

It may be relevant to investigate whether infection, as observed in this clinical case, associated with the retained root complex after coronectomy of an MTM, directly contributes to further root migration. While physiological inflammation is essential for soft tissue and bone healing, persistent infection may disrupt this balance, impair normal bone remodeling, and contribute to pathological changes in the tissues surrounding the retained root complex. As a result, the root complex may migrate more readily in that direction [11].

The relationship between inflammation and healing following coronectomy deserves further consideration. Inflammation is not only a response to infection but also an essential component of both soft tissue and bone healing. During the early healing phase, inflammatory cells release cytokines and growth factors that promote angiogenesis, fibroblast proliferation, and tissue repair [28]. Similarly, inflammatory mediators play a central role in bone remodeling by regulating osteoclast and osteoblast activity, which is necessary for the restoration of normal bone architecture after dentoalveolar surgery [25, 26]. Comparable mechanisms have been described in extraction socket healing, where a controlled inflammatory response is required for subsequent bone regeneration [27]. In the present case, physiological bone remodeling may have contributed to the observed root migration, whereas the later development of infection may have altered local bone turnover and influenced the behavior of the retained root complex. Further studies are needed to clarify the relationship between inflammation, bone remodeling, root migration, and long‐term outcomes following coronectomy.

Despite the risk of complications associated with residual roots, some studies have reported that pain and alveolitis occur at similar or even lower rates after coronectomy compared with complete extraction, likely because of reduced socket trauma and smaller wound sizes [1, 2, 8]. Kang et al. [2], in a RCT involving 110 MTMs (*N* = 55), found that the duration of postoperative pain was significantly shorter in the coronectomy group (2.61 ± 1.95 days) than in the extraction group (3.40 ± 1.55 days). Furthermore, Hamad [1] reported a substantially lower incidence of dry socket in the coronectomy group (4.5%) compared with the extraction group (10.5%).

The patient of this case report was a 41‐year‐old female. According to the previous studies mentioned earlier, age and sex are well‐documented predictors of root migration. On the other hand, younger individuals appear to present more extensive migration of the remaining root complex left after coronectomy, likely due to higher metabolic activity in the periodontal ligament and alveolar bone, while older individuals exhibit reduced migration because of fibrosis and bone sclerosis [3, 4, 15, 17, 18]. Leung and Cheung [17] and Kohara et al. [3] describe that female patients also exhibit more extensive root migration than males, which has been suggested to be attributed to lower bone density in the mandible and more conical root morphology in MTMs [3, 4, 7, 15]. It is also reported that morphology affects migration patterns. Conical or single‐rooted molars tend to migrate more and more rapidly than multirooted or club‐shaped roots, likely due to reduced resistance and less mechanical interlocking with surrounding bone [2, 11, 15, 18]. In our study subject, this was not the case since the tooth presented a multirooted morphology. Additionally, shallow impactions and the presence of space between the MTM and the mandibular ramus were associated with greater migration [4]. Conversely, deeper or distoangular impactions tend to show less movement [2, 4, 11, 15, 18].

Radiographic follow‐up protocols remain variable, although some studies suggest reassessment at 6 months and every 2 years thereafter, especially if symptoms such as root exposure or discomfort develop [3, 8, 11, 15]. CBCT imaging could offer a more accurate assessment of root migration and IAN proximity than conventional 2D radiography, enabling safer planning for delayed root removal if necessary [2, 3, 7, 9, 11].

As a secondary finding of this case report, radiographic follow‐up demonstrated favorable bone formation distal to tooth 37 despite the development of infection and the patient's age being over 30 years (Figure 11). However, because periodontal probing measurements were not recorded at the final follow‐up examination, the long‐term periodontal status of the distal aspect of the second molar cannot be objectively assessed. Further studies could be of interest to evaluate the long‐term periodontal outcomes of second molars following coronectomy and subsequent root removal.

The available evidence regarding the cost‐effectiveness of coronectomy remains limited, particularly when long‐term follow‐up and potential reinterventions are considered. Pitros et al. [12] performed a cost–benefit analysis based on direct treatment costs and reported that coronectomy was less expensive than surgical extraction, primarily because of the lower incidence of inferior alveolar nerve injury (12%). However, this finding was dependent on the diagnostic protocol used, as the routine use of CBCT increased the overall treatment cost. The authors also highlighted the uncertainty surrounding the long‐term need for reintervention, which may influence the overall economic outcome of the procedure. Consequently, the true cost‐effectiveness of coronectomy remains unclear and should be evaluated in future studies incorporating long‐term clinical outcomes, reintervention rates, imaging requirements, and patient‐reported measures of quality of life. The value assigned to the prevention of inferior alveolar nerve injury is also likely to play a significant role in such analyses.

## Author Contributions

**Vito Marquez Rocco:** conceptualization, data curation, formal analysis, investigation, methodology, project administration, visualization, writing – original draft, writing – review and editing. **Andreas Thor:** conceptualization, formal analysis, investigation, methodology, supervision, writing – review and editing. **Abdullah A. Hashim:** formal analysis, investigation, visualization, writing – original draft. **Dominique Bellerud:** visualization, writing – original draft.

## Funding

The authors have nothing to report.

## Ethics Statement

Ethical approval for case reports is not required by the institutional or national research ethics committee according to local regulations.

## Consent

Written informed consent was obtained from the patient for publication of this case report and any accompanying images both in English and Swedish.

## Conflicts of Interest

The authors declare no conflicts of interest.

## Data Availability

All relevant data generated or analyzed during this case report are included in this published article. Additional information is available from the corresponding author upon reasonable request.
